# Antiferromagnetic half-skyrmions electrically generated and controlled at room temperature

**DOI:** 10.1038/s41565-023-01386-3

**Published:** 2023-05-08

**Authors:** O. J. Amin, S. F. Poole, S. Reimers, L. X. Barton, A. Dal Din, F. Maccherozzi, S. S. Dhesi, V. Novák, F. Krizek, J. S. Chauhan, R. P. Campion, A. W. Rushforth, T. Jungwirth, O. A. Tretiakov, K. W. Edmonds, P. Wadley

**Affiliations:** 1 School of Physics and Astronomy, University of Nottingham, Nottingham, UK; 2 Diamond Light Source, Chilton, UK; 3 Institut für Physik, Johannes Gutenberg Universität Mainz, Mainz, Germany; 4 Institute of Physics, Czech Academy of Sciences, Prague, Czech Republic; 5 School of Physics, The University of New South Wales, Sydney, New South Wales Australia

**Keywords:** Spintronics, Magnetic properties and materials

## Abstract

Topologically protected magnetic textures are promising candidates for information carriers in future memory devices, as they can be efficiently propelled at very high velocities using current-induced spin torques. These textures—nanoscale whirls in the magnetic order—include skyrmions, half-skyrmions (merons) and their antiparticles. Antiferromagnets have been shown to host versions of these textures that have high potential for terahertz dynamics, deflection-free motion and improved size scaling due to the absence of stray field. Here we show that topological spin textures, merons and antimerons, can be generated at room temperature and reversibly moved using electrical pulses in thin-film CuMnAs, a semimetallic antiferromagnet that is a testbed system for spintronic applications. The merons and antimerons are localized on 180° domain walls, and move in the direction of the current pulses. The electrical generation and manipulation of antiferromagnetic merons is a crucial step towards realizing the full potential of antiferromagnetic thin films as active components in high-density, high-speed magnetic memory devices.

## Abstract (web summary)

The implementation of topological antiferromagnetic vortices in information storage devices requires an efficient method of nucleation and a way to control their movement. Here the authors find CuMnAs to be a suitable electrically conducting antiferromagnet host material for topological spin textures.

## Main

The defining feature of a topological texture is a non-zero winding number, also called topological charge. In magnetic systems, this is a measure of the integer number of times the order parameter wraps around a unit sphere (Bloch sphere) when integrated over the texture volume. Magnetic textures with different winding numbers are topologically distinct and cannot be easily changed from one topological state to another. This provides strong protection against perturbation and prevents their collapse even at ultrasmall sizes^1^. In ferromagnetic (FM) thin films, where the magnetization defines the topological charge, *Q*, skyrmions (*Q* = ±1) and merons (*Q* = ±1/2) have been generated and controlled using external fields and current-induced spin torques^2–9^. However, their implementation in practical devices has been limited by the presence of gyrotropic forces, originating from their topology, that cause an unwanted transverse deflection of their current-driven motion^10^.

**Fig. 1:**
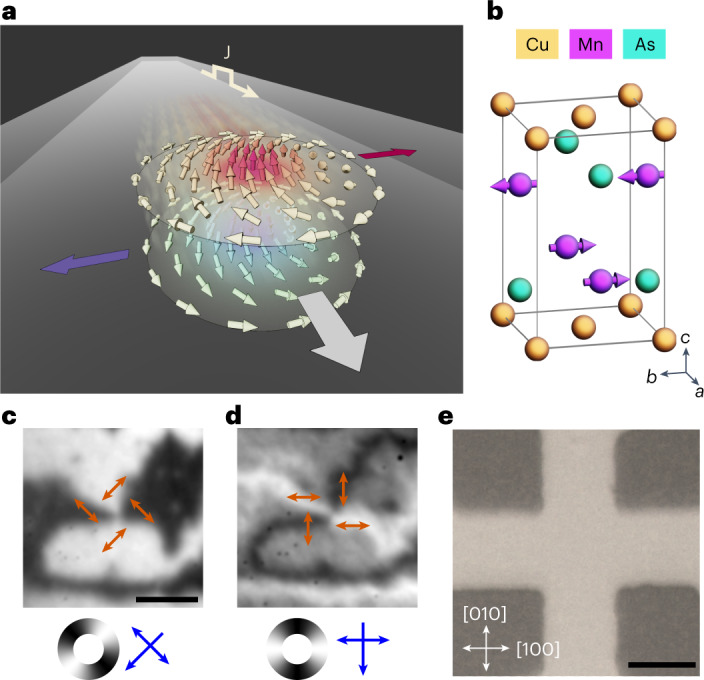
AF textures in CuMnAs. **a**, Spin structure and force acting on an AF Bloch-type meron under an applied current pulse **J**. **b**, Unit cell and magnetic structure of CuMnAs. **c**,**d**, XMLD–PEEM images of a vortex structure in CuMnAs. The blue single- and double-headed arrows indicate the X-ray incidence and polarization vectors, while the colour wheels and red double-headed arrows indicate the spin axis orientation inferred from the XMLD contrast. The scale bar corresponds to 1 μm. **e**, Optical image of the device structure used for electrical pulsing. The spatial scale bar corresponds to 10 μm.

This effect is alleviated in antiferromagnets due to their compensated FM sublattices. The antiferromagnetic (AF) order parameter, called the Néel vector, is given by **L** = **M**_1_ − **M**_2_ in collinear antiferromagnets, where **M**_1_ and **M**_2_ are antiparallel sublattice magnetizations. Analogous to the magnetization in ferromagnets, the Néel vector defines the Néel topological charge, *Q*_N_, of the AF spin texture^11,12^. However, gyrotropic forces, ${\mathbf{F}}_{{{{\rm{gyro}}}}}^{(k)}={G}^{(k)}\hat{\mathbf{z}}\times {{{\mathbf{J}}}}$, induced by the current density, **J**, depend on the gyrocoupling constant *G* = 4π*Q*^(*k*)^, which is a function of the sublattice topological charge, *Q*^(*k*)^. When integrated over the texture volume the gyrotropic forces fully compensate, resulting in a remaining generalized drag force, **F**_drag_ = *Γβ***J**, in the direction of the current, where *β* is the sum of the current-induced spin torques acting on the AF topological texture and *Γ* is the damping parameter^13^. The dynamics of the AF topological texture under the action of current-induced spin torques is described by the generalized Thiele equation^14–16^,

1 $${F}^{i}={{{{\mathcal{M}}}}}^{ij}{\ddot{b}}_{j}+\alpha {{{\varGamma }}}^{ij}{\dot{b}}_{j},$$

where the *b*_*j*_ are the collective coordinates of the topological spin structure, ${{{{\mathcal{M}}}}}^{ij}$ is the mass tensor and *αΓ*^*ij*^ characterizes the viscous friction. The AF topological texture is propelled by this force at terminal velocity **v**_∥_ = *β***J**, parallel to the current direction, as shown schematically in Fig. 1a.

The incentive for finding materials that host AF topological textures has led to their experimental observation in several systems: complex synthetic AF heterostructures^17^, FM/AF bilayers^18–20^ and most recently pure AF materials, MnSc_2_S_4_^21^ and α-Fe_2_O_3_^22^. In MnSc_2_S_4_ a fractional AF skyrmion lattice is formed at low temperature, with the application of a large magnetic field. In α-Fe_2_O_3_, AF merons and antimerons are stabilized at room temperature by thermal cycling through a Morin transition point. Both examples represent an important advancement for AF skyrmionics, but the nucleation methods are impractical for integrable devices. It has also yet to be shown that, once generated, topological AF textures can be controllably moved. Here, we bridge this gap by showing that AF merons and antimerons can be electrically generated and controlled at room temperature in a conducting AF material, CuMnAs. CuMnAs has the required crystal symmetries to host current-induced Néel spin orbit torques, which can efficiently manipulate the AF order^23–26^. This makes it an ideal candidate material for antiferromagnet-based spintronic devices^27–29^.

## Imaging AF textures in CuMnAs

Our experiments are performed on tetragonal phase CuMnAs (space group *P*4/*nmm*), epitaxially grown on a lattice-matched GaP(001) substrate^30^. Below the Néel temperature of 480 K, the Mn atoms form two magnetic sublattices, which are stacked vertically in the crystallographic *c* axis (Fig. 1b). The magnetic easy axis lies in the *ab* plane due to magnetocrystalline anisotropy. The precise behaviour of the magnetic anisotropy is strongly influenced by the interface with the substrate and, depending on film thickness, can be tuned between in-plane uniaxial and biaxial anisotropies^31^.

To resolve the magnetic structure we used X-ray magnetic linear dichroism (XMLD) combined with photoemission electron microscopy (PEEM). Figure 1c,d shows an image of an AF vortex structure in CuMnAs. The colour wheels and red double-headed arrows in Fig. 1c,d indicate the local spin axis orientation. This is determined from the measured XMLD by comparison with previously published XMLD spectra from CuMnAs films with known spin orientations^32,33^. With the incident X-ray polarization along one of the biaxial easy axes (Fig. 1c), the 0° and 90° AF domains appear as dark and light regions, respectively. When the polarization is at 45° to the easy axes, the contrast is largest at the AF domain walls (AFDWs), which appear either dark or light depending on the orientation of the spin at the domain wall centre (Fig. 1d). The AF vortex is identified by the dark–light–dark–light pattern of the AFDWs along a circular path. While it is not possible to distinguish the sign of **L** by XMLD, the chirality of the vortex structure depends only on the spin axis rotation, determinable from the two X-ray configurations. The vortex core size is below the measurement resolution of ≈40 nm, as discussed in Supplementary Section 2.

## Generation of merons and antimerons using electrical pulses

**Fig. 2:**
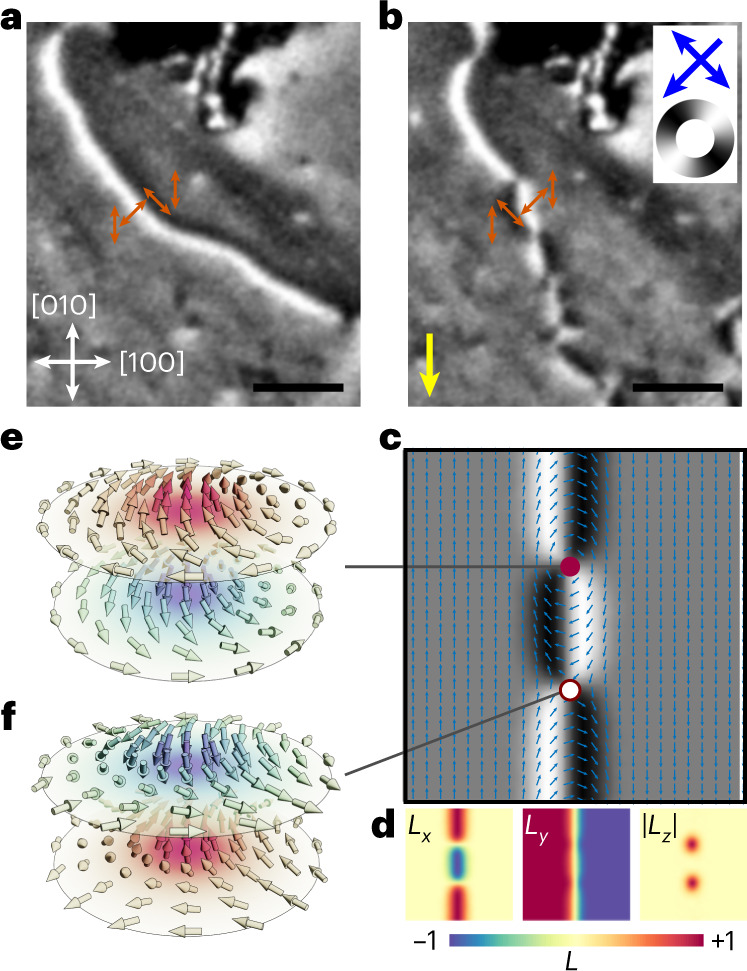
Generation of AF meron–antimeron pairs using an electrical pulse. **a**, XMLD–PEEM image of a 180° AFDW located in the top arm of the device. White and black contrast corresponds to Néel vector orientation perpendicular and parallel to the X-ray linear polarization (blue double-headed arrow in top right of **b**), respectively. The spin axis variation across the AFDW width is depicted by red arrows. The spatial scale bar corresponds to 600 nm. **b**, The AFDW after applying a 1 ms electrical pulse with 21 V (1.2 × 10^7^ A cm^−2^) amplitude along the [0$\bar{1}$0] CuMnAs crystal direction (yellow arrow), showing sections of reversed chirality. The chirality changes are associated with AF vortices and antivortices. **c**,**d**, Simulated XMLD–PEEM image and Néel vector heatmaps for an AFDW showing chirality reversal. The colour bar represents the magnitude of the Néel vector along each component (*L*_*x*,*y*,*z*_). **e**,**f**, Characteristic Bloch-type meron (**e**) and antimeron (**f**) with out-of-plane core spin component, located at positions highlighted by red and white filled circles in **c**. Source data

To investigate the current-induced generation and manipulation of topological spin structures, we fabricated a four-arm cross device with 10 μm arm width from a 50 nm layer of CuMnAs (Fig. 1e). In the patterned sample, the magnetic anisotropy is uniaxial, with domains oriented along the [010] crystalline axis. Figure 2a shows an XMLD–PEEM image of a 180° AFDW identified in one of the arms of the device. Incident X-rays have linear polarization vector along the [1$\bar{1}$0] CuMnAs crystal axis. The variation in **L** across the AFDW width is resolved as white and black contrast, corresponding to in-plane magnetic moments aligned perpendicular and parallel to the X-ray polarization, respectively, as indicated by the colour wheel in the top right corner of Fig. 2b.

A 1 ms electrical pulse, with amplitude 21 V (corresponding to a current density of 1.2 × 10^7^ A cm^−2^) was applied along the [010] CuMnAs axis of the device. Figure 2b shows the micromagnetic structure of the AFDW after the pulse was applied. Sections of the AFDW have the order of contrast reversed from white/black to black/white, corresponding to a reversal of chirality.

Monte Carlo methods with magnetic anisotropy parameters typical for thin-film CuMnAs^9,34^ were used to simulate the structure of the AFDW. The simulated dichroism image with overlaid top sublattice magnetization is shown in Fig. 2c. Topological spin textures are seen at the AFDW chirality reversal points, with the positions of their cores highlighted by filled circles. At the position of the red filled circle, the spin texture has the characteristic structure of a Bloch-type meron: spins along its diameter form a Bloch-type 180° domain wall. The spin texture at the position of the white filled circle is an antimeron. There are two directions along which the diameter forms Bloch-type 180° domain walls, and two that form Néel-type 180° domain walls. The *L*_*x*_, *L*_*y*_ and ∣*L*_*z*_∣ Néel vector components are plotted as heatmaps in Fig. 2d. Most notably, ∣*L*_*z*_∣ shows an out-of-plane spin orientation at the core of the vortex—as is required for topologically non-trivial textures. The full three-dimensional spin structures of the AF meron and antimeron are shown in Fig. 2e,f.

## Electrical control of generated meron–antimeron pairs

**Fig. 3:**
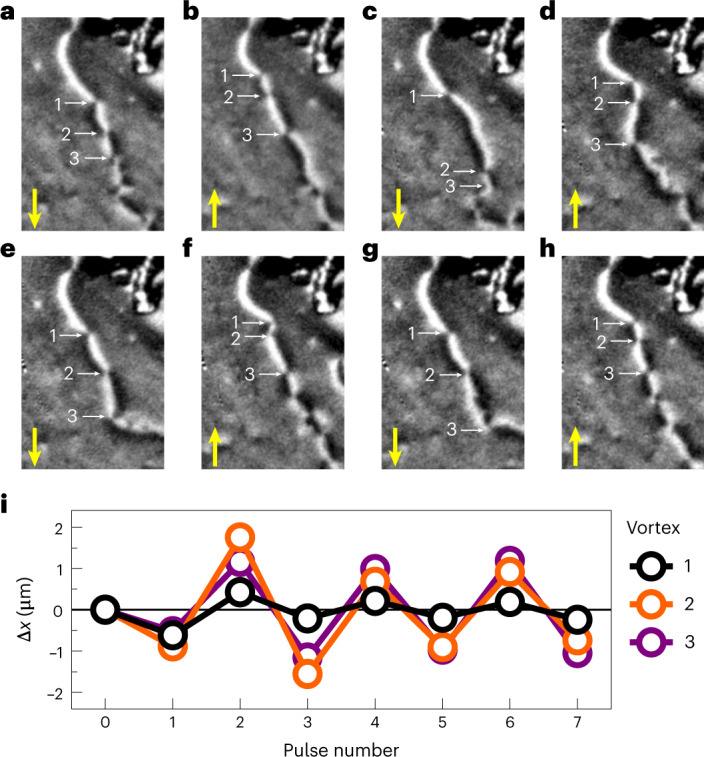
Movement of meron–antimeron pairs using electrical pulses. **a**–**h**, XMLD–PEEM images of the 180° AFDW after applying successive 1 ms, 21 V electrical pulses in the directions indicated by the yellow arrows. The width of each image corresponds to 1.2 μm. **i**, The average displacement of vortices 1 to 3 indicated in **a**, measured after each pulse. Source data

Subsequent 1 ms electrical pulses, with 21 V amplitude, were applied in a sequence of alternating polarity along the [010] CuMnAs axis. Figure 3 shows XMLD–PEEM images of the 180° AFDW after each pulse in the sequence. Yellow arrows show the current direction of the applied pulse. The AFDW state shown in Fig. 3a corresponds to the state shown in Fig. 2b. After successive pulses of alternating polarity, shown in Fig. 3b–h, the points of AFDW chirality reversal, where merons and antimerons are localized, are seen to move collectively in the direction of the applied pulse. For each image, the same meron–antimeron pair can be identified as the white/black, black/white (and vice versa) contrast reversal, indicating a change in the AFDW chirality, which necessitates the type of topological vortex at that point (Supplementary Section 1). The displacement, Δ*x*, of three merons indicated in Fig. 3a, along the [0$\bar{1}$0] direction, collinear with the pulse direction, is shown in Fig. 3i for the sequence of eight alternating polarity pulses. Their movement follows a reversible, repeatable pattern dependent on the pulse polarity. Thermal effects are precluded from causing the reversible motion, as these are independent of the pulse direction.

We note that the current pulses used in Fig. 3 are of the same amplitude and duration as the pulses used to generate the spin textures in Fig. 2a,b. Therefore, we cannot entirely rule out annihilation/recreation of spin textures at these current amplitudes. However, the repeatable back-and-forth behaviour with alternate pulses, shown in Fig. 3i, is succinctly explained by current-driven motion between pinning sites, created by crystal defects and local strains, which determine the stable positions for the meron–antimeron pairs. In Supplementary Section 4, we present experimental data of current-induced domain wall motion at current amplitudes below the threshold for generation.

## Observation of isolated meron–antimeron pairs on AFDW loops

**Fig. 4:**
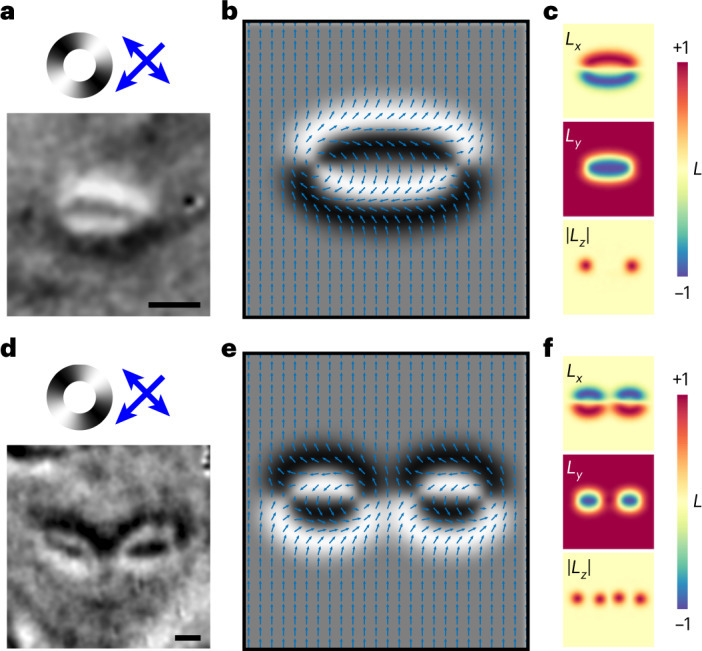
Isolated meron–antimeron pairs localized at the points of chirality reversal on 180° AFDW loops. **a**,**d**, XMLD–PEEM images of a single 180° AFDW loop (**a**) and double 180° AFDW loops (**d**). Spatial scale bars correspond to 350 nm. **b**,**c**,**e**,**f**, Simulated XMLD–PEEM images (**b**,**e**) and Néel vector heatmaps (**c**,**f**) for both structures showing meron–antimeron pairs situated at points of AFDW chirality reversal. The colour bar represents the magnitude of the Néel vector along each component (*L*_*x*,*y*,*z*_).

As well as the electrical generation and control of meron–antimeron pairs along a 180° AFDW, we observed, in distinct regions of the device, the formation of isolated pairs on 180° AFDW loops. Figure 4a,d shows two XMLD–PEEM images of example 180° AFDW loops. On each loop there exist two points of chirality reversal corresponding to a meron and antimeron pair. This is revealed in the micromagnetic simulations of such structures shown in Fig. 4b,e. In the case of two adjacent AFDW loops, shown in Fig. 4d, a meron and antimeron in close proximity form a bimeron, depending on the polarity of each structure. Further examples of observed isolated meron–antimeron pairs are presented in Supplementary Fig. 7. We show evidence of isolated pair formation during a sequence of electrical pulses and their resistance to motion at pulse amplitudes below the nucleation threshold.

## Proposed mechanism for generating and controlling AF meron–antimeron pairs

To generate a topologically non-trivial meron–antimeron pair from vacuum requires the supply of a critical energy within a short enough time interval so as not to be dissipated through damping in the system^35^. In the exchange-dominated limit, this critical energy is defined by a topological invariant, 8π∣*Q*_N_∣*At*, where *A* is the AF exchange stiffness, *t* is the sample thickness and *Q*_N_ is the Néel topological charge of the nucleated spin structure. We inject energy into our system by applying electrical pulses, which induce meron–antimeron pair generation through the combined action of thermal and spin-torque effects.

If a topologically trivial meron–antimeron pair is generated with equal core polarity, then *Q*_N_ = 0, leading to zero nucleation threshold energy. However, the potential barrier separating such a pair from annihilation is small, leading to a low probability of survival^36^. Instead, the topologically non-trivial case of a bimeronic pair, where *Q*_N_ = ±1, has a larger separating barrier and is the more stable state. Thus, although the meron–antimeron core polarity is not determinable in XMLD–PEEM, we attribute the observed meron–antimeron pairs as topologically non-trivial states.

In our system, meron–antimeron pair generation is localized on 180° AFDWs as this is most energetically favourable, confirmed by Monte Carlo simulations shown in Supplementary Section 3. AFDWs are topologically non-trivial in one dimension and are metastable states that can readily host two-dimensional topological spin structures. Nucleation of a meron–antimeron pair is a stochastic event, with probability proportional to the length of available AFDW. Thus, after the first instance, the probability (energy) for further pair nucleation is reduced (increased).

Applying pulses with alternating polarity causes the meron–antimeron pairs to move back and forth between pinning sites, remaining localized on the AFDW. As the XMLD–PEEM images are taken after each pulse is applied, it is not possible to distinguish between current-driven displacement and annihilation/recreation at new pinning sites. However, the results presented in Fig. 3 show that the merons are displaced in the current direction, and exhibit a repeatable polarity-dependent motion during the sequence of eight pulses. This observed phenomenon is well explained by a model based on current-induced spin torques as the driving mechanism. As highlighted in equation (1), all spin-torque contributions force the merons in the current direction independent of their topological charge^14–16^.

The scenario presented in Fig. 3 represents a near-optimum geometry for observing current-induced meron and antimeron displacement, because 180° AFDW motion is minimized. The pulse direction is collinear with both the spin axis and the AFDW length, which satisfies the condition that no AFDW movement driven by Néel spin–orbit torque should occur^27,37^. Away from this geometry, the movement of merons and antimerons is also expected, but can be complicated by additional AFDW displacement, as shown in Supplementary Section 4. However, it is also demonstrated that, even away from the optimum geometries, meron–antimeron motion along a static AFDW can occur given sufficient domain wall pinning, such as along a structural defect line (Supplementary Fig. 6).

## Conclusions

Overall, the results presented demonstrate CuMnAs to be a suitable electrically conducting AF host material for topological spin textures. Efficient generation and control of AF merons and antimerons using electrical pulses paves the way towards realizing practical antiferromagnet-based solitonic devices. The role of magnetic anisotropy in stable meron size can be explored by tuning the CuMnAs epilayer thickness^31^, and the controlled fabrication of structural pinning sites^33^ may be used to selectively determine distances between which merons and antimerons move. This could lead to the development of novel device architectures, combining constricted channels to nucleate isolated meron–antimeron pairs^4^ that may be used to carry information on racetrack memories^38–41^.

## Methods

### Sample fabrication

The 50 nm CuMnAs film used for this study was grown by molecular beam epitaxy on a GaP buffer layer on a GaP(001) substrate, using the procedures described in ref. ^30^. The film was capped with a 3 nm Al film to prevent surface oxidation. The device structure shown in Fig. 1e, with device arms aligned along the [100] and [010] CuMnAs crystal axes, was fabricated using optical lithography followed by ion etching.

### PEEM imaging and electrical pulsing

The PEEM measurements were performed on beamline I06 at Diamond Light Source. The X-ray beam was incident at a grazing angle of 16°, with the X-ray linear polarization in the plane of the film. The asymmetry, ${{\varDelta }}=({I}_{{E}_{1}}-{I}_{{E}_{2}})/({I}_{{E}_{1}}+{I}_{{E}_{2}})$, between images obtained at the Mn L_3_ absorption peak (*E*_1_) and 0.9 eV below the L_3_ peak (*E*_2_) provides a map of the local spin axis with 30 nm spatial resolution. The sample was mounted on a cartridge with four wire-bonded electrical contacts allowing electrical pulses to be applied in situ within the PEEM vacuum chamber. After each pulse, XMLD–PEEM images of device regions were taken to map changes to the micromagnetic structures. The time between applying a pulse and acquiring an image exceeded 120 s, thus only non-transient changes to the magnetic state were observed.

### Micromagnetic simulations

We perform Monte Carlo simulations of magnetic textures observed in CuMnAs on a 51 × 51 × 2 system of spins. The system has periodic boundary conditions in the first (*x*) and third (*z*) dimensions, and closed boundaries in the second (*y*). Each layer of the system (in *z*) corresponds to a magnetic sublattice, with AF coupling between the layers. The total energy of the system is defined as the sum of the exchange energy, the out-of-plane magnetic anisotropy and the in-plane magnetic anisotropy. Spins have full rotational degrees of freedom with out-of-plane polar angle *θ* and in-plane azimuthal angle *ϕ*. The system is initiated with in-plane textures and allowed to settle for *N* × 10^6^ iterations.

## Online content

Any methods, additional references, Nature Portfolio reporting summaries, source data, extended data, supplementary information, acknowledgements, peer review information; details of author contributions and competing interests; and statements of data and code availability are available at 10.1038/s41565-023-01386-3.

## Supplementary information

Supplementary Information Supplementary Sections 1–4 and Figs. 1–7.

## Data Availability

The data supporting the findings of this study are available from the corresponding author upon reasonable request. Source data are provided with this paper.

## Source data

Source Data Fig. 2 Unprocessed PEEM images of AF meron–antimeron pair generation.
Source Data Fig. 3 Unprocessed PEEM images of current-driven meron–antimeron displacement, and corresponding statistical source data.

## Code availability

The code used to generate simulation results that support the findings of this study is publicly available at https://github.com/oamin/AF-merons.
