# Supplementary material for: Antiferromagnetic half-skyrmions electrically generated and controlled at room temperature
Source: Nat Nanotechnol. 2023 May 8;18(8):849–53. doi: 10.1038/s41565-023-01386-3 (PMC10427425; doi:10.1038/s41565-023-01386-3)
Supplement: Supplementary file 1 — Supplementary Sections 1–4 and Figs. 1–7. [file 41565_2023_1386_MOESM1_ESM.pdf]

nature nanotechnology

Article

https://doi.org/10.1038/s41565-023-01386-3

Antiferromagnetic half-skyrmions electrically generated and controlled at room temperature

In the format provided by the
authors and unedited

Contents

S1 Determining the spin texture chirality using XMLD-PEEM **2**

S2 Estimating the size of the meron core **4**

S3 Nucleation energy of a meron-antimeron pair localised on/away from a 180° domain wall **6**

S4 Supplementary measurements of current-induced generation and motion of spin textures **9**

S1 Determining the spin texture chirality using XMLD-PEEM

Figure S1 shows XMLD-PEEM images of the antiferromagnetic spin texture in a CuMnAs device, for x-ray polarization along the $[1\bar{1}0]$ and [010] crystal axes. A meron-like structure in a 180° domain wall, and a set of sub-micron sized closed-loop domain walls, are highlighted and shown on an expanded scale in Fig. S1c,d and Fig. S1e,f, respectively. The 180° wall appears as a dark line for x-ray polarization along [010], whereas for polarization along $[1\bar{1}0]$ adjacent dark/light lines are observed. Similarly, the closed-loop walls appear as dark circles for polarization along [010], and show an alternating dark-light variation for polarization along $[1\bar{1}0]$. The images with polarization along $[1\bar{1}0]$ are comparable to the simulated images shown in Fig. 2c and Fig. 4e of the main paper.

The XMLD amplitude in CuMnAs varies as $A \cos 2\theta$, where θ is the angle between the x-ray polarization and the local magnetic moment orientation, and A depends on the orientation of the x-ray polarization with respect to the crystalline axes. The pre-factor A has been determined in previous measurements of CuMnAs films with known orientation of the spin axis [1]. The corresponding dependence of the XMLD-PEEM contrast is indicated by the colour wheels in Fig. S1: light and dark regions for x-ray polarization along the $[1\bar{1}0]$ axis correspond to local magnetic moments oriented along [110] and $[1\bar{1}0]$ respectively, whereas for polarization along [010] the light and dark regions correspond to moments along [010] and [100] respectively.

With the axis-dependence of the XMLD-PEEM contrast established, the chirality of the spin-rotation across the domain wall can be determined from the two x-ray configurations, if we assume that the spins rotate gradually across the domain wall. This is illustrated by the double-headed arrows in Fig. S1d,e, where the local moments for the two magnetic sublattices are indicated in different colours. From this it can be seen that the spin texture in Fig. S1c,d is an antimeron, and the reversal of contrast in the $[1\bar{1}0]$ image corresponds to a reversal of the domain wall chirality.

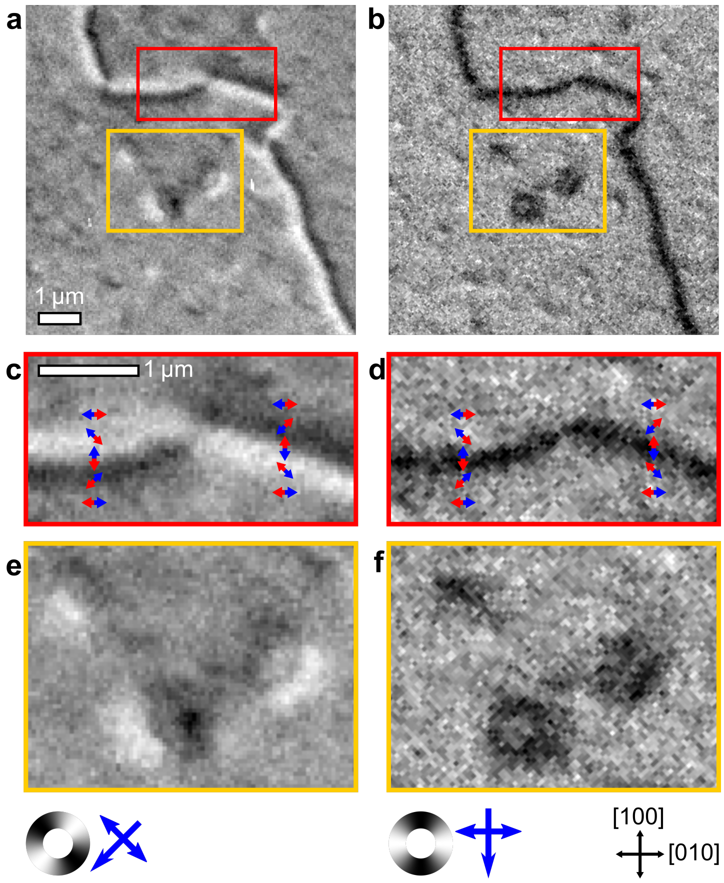

Figure S1: Determining the spin texture chirality from XMLD-PEEM. **a**,**b** Images of an extended 180° domain wall and submicron-scale closed loop domain walls for x-ray polarization along $[1\bar{1}0]$ (a) and [010] (b). **c**,**d** and **e**,**f** are expanded views of the regions shown in the red and orange rectangles in **a** and **b**, respectively. The double-headed arrows in **c** and **d** indicate direction of the sublattice magnetizations, as determined by the known angular dependence of the XMLD signal indicated by the colour wheels. The scale bar in each image corresponds to 1 µm.

S2 Estimating the size of the meron core

Although the direction of the meron core spin cannot be distinguished using XMLD-PEEM, the presence of an out-of-plane spin component may be identified by tilting the x-ray polarization out of the plane, if the core is comparable to or larger than the instrument resolution [2]. In Fig. S1a-c, we compare XMLD-PEEM images obtained with x-ray polarization in-plane and out-of-plane. No features are observed above the background at the position of the meron in the out-of-plane polarization image (Fig. S1c). Therefore, the meron core cannot be distinguished within the instrument resolution of ≈40 nm for the present samples.

The size of the core is given approximately by $\sqrt{A/K_{\perp}}$ where $K_{\perp}$ is the out-of-plane anisotropy energy density, and A is the exchange stiffness. In Ref. [3], $K_{\perp}$ was calculated using relativistic density functional theory to be 127 µeV per Mn. The exchange stiffness can be estimated from the measured ≈200 nm width of the the 180° domain walls, which corresponds approximately to $\sqrt{A/K_{\parallel}}$ where $K_{\parallel}$ is the in-plane anisotropy energy density. $K_{\parallel}$ could not be determined within the resolution of the DFT calculations in Ref. [2], but was estimated by measurements of the spin-flop field to be around 0.005 T [4], which for a local moment of 3.6 μ_{B} corresponds to 0.5 µeV per Mn. Hence, the size of the core is expected to be of the order of 10 nm, which significantly smaller than the resolution for the PEEM instrument. Therefore, the core is expected to be unresolvable in this experiment, consistent with our measurements.

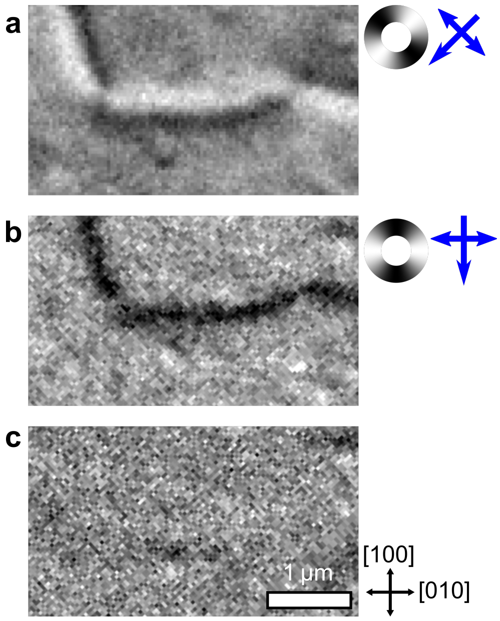

Figure S2: Section of domain wall with two points of chirality reversal (meron and antimeron) imaged with x-ray polarisation along the **a**, in-plane $[1\bar{1}0]$ axis, and **b**, [010] axis, and **c**, out-of-plane [001] axis.

S3 Nucleation energy of a meron-antimeron pair localised on/away from a 180° domain wall

The energy for nucleation of a meron-antimeron pair was studied for two systems (with and without a 180° domain wall) using Monte Carlo simulations outlined in the Methods section of the main text. The system consists of $100 \times 100 \times 2$ spins, antiferromagnetically coupled along the out-of-plane axis. In the first instance (**a**), a meron, with topological charge $Q \approx 0.5$, was initialised in a uniform domain and allowed to relax for 10^{10} iterations per spin. The same procedure was repeated with the inclusion of a 180° AFDW, upon which the meron was localised (**b**).

As the systems relax, the point of highest energy along the perimeter of the initial meron state forms an antimeron with opposite polarity, so that the meron-antimeron pair state forms a topologically non-trivial bimeron, with Q approaching 1. Figures S3a and b show vector plots of the relaxed states for the respective cases, with corresponding colour maps showing the x, y and z Néel vector components. The bottom plot of Fig. S3c shows the system topological charge during this relaxation. In both cases, the topological charge approaches 1, corresponding to a topologically non-trivial meron-antimeron pair, or bimeron state.

The system energy was calculated for each scenario, **a** and **b**, after subtracting the energy of the corresponding background state (a uniform domain and unperturbed 180° AFDW, respectively). The energy after subtraction is plotted as the system relaxes in the top plot of Fig. S3c. The meron-antimeron pair localised on the domain wall is the lower energy state (orange line), and thus the more likely scenario to occur, consistent with the experimental observations.

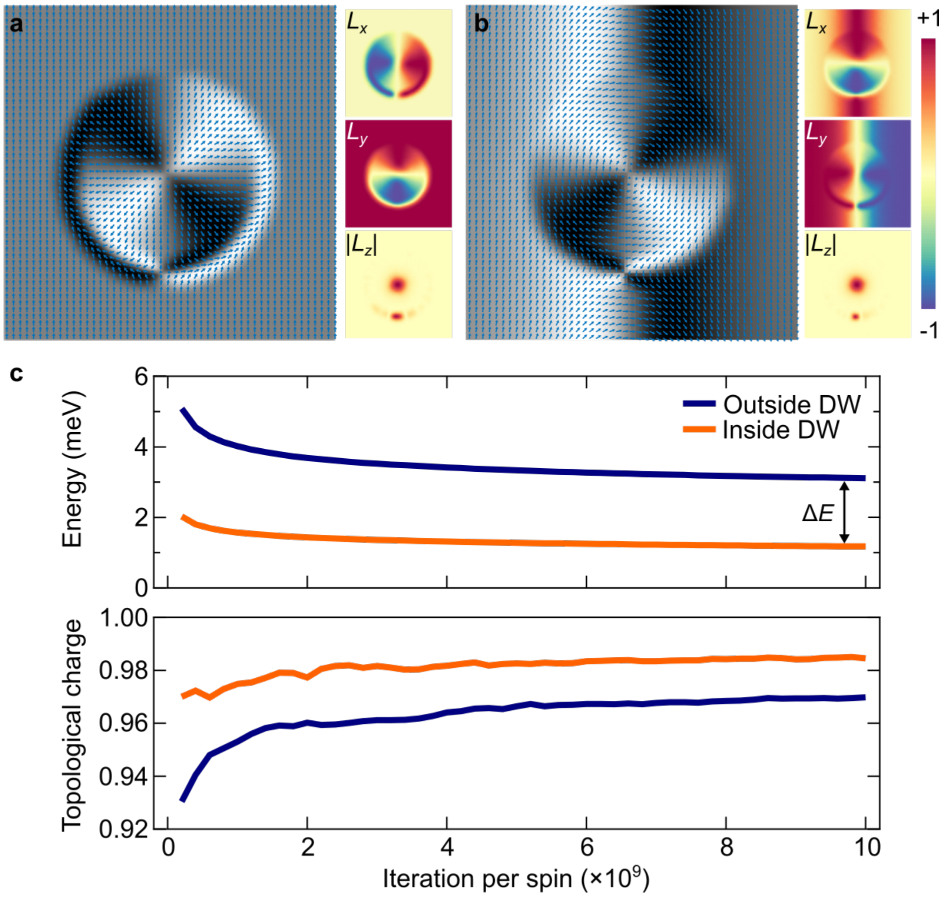

Figure S3: Simulated meron-antimeron pair formation localised **a**, in a uniform domain, and **b**, on a 180° domain wall. **c**, The system energy and topological charge, measured over 10^{10} iterations per spin.

S4 Supplementary measurements of current-induced generation and motion of spin textures

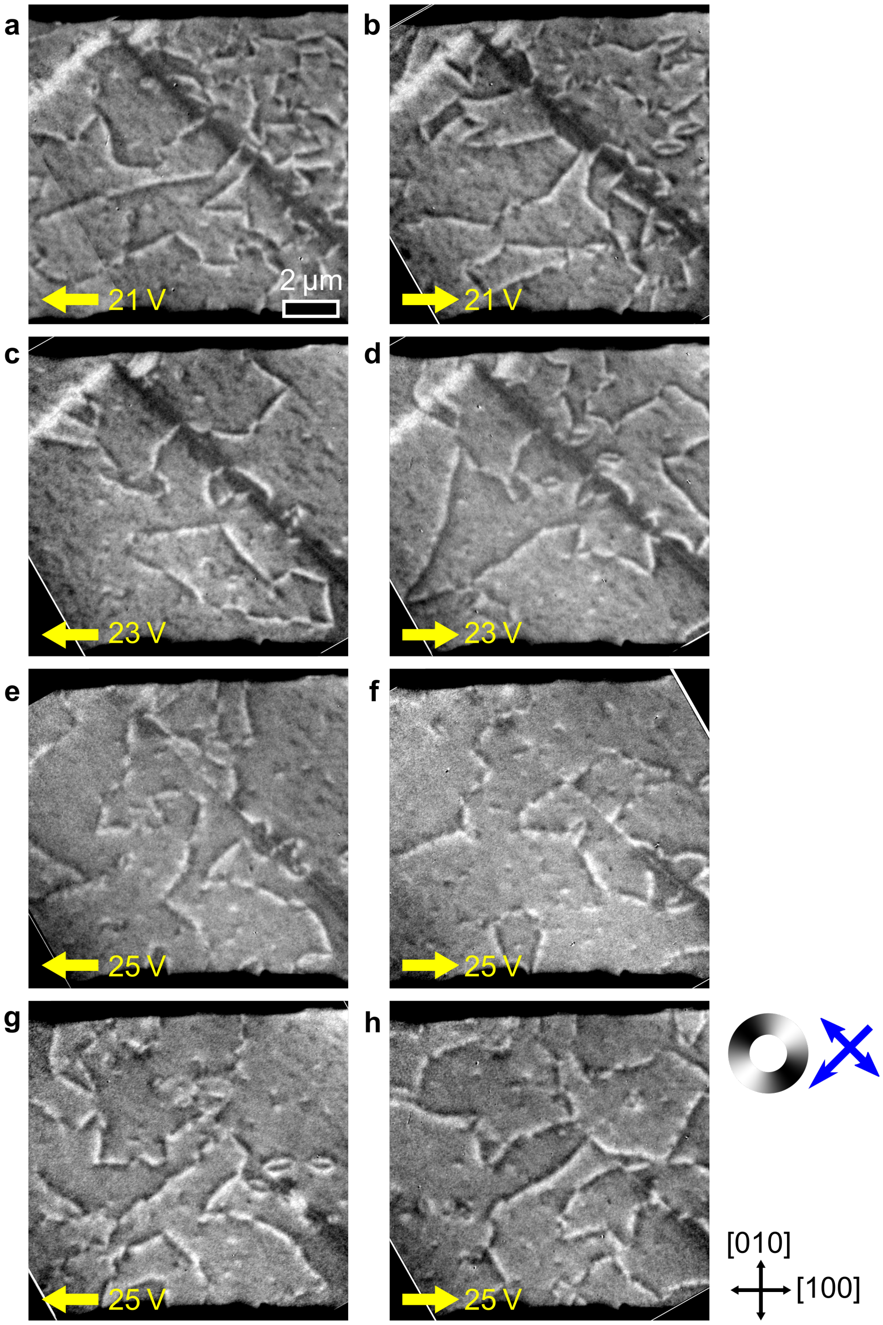

Figure S4: XMLD-PEEM images after current pulses applied along the [100] arm of the 10 μm CuMnAs device. The x-ray polarization is along the [1$\bar{1}$0] axis.

In this section we present further experimental results demonstrating the generation and manipulation of merons and antimerons in CuMnAs devices. Figure S4 shows the effect of pulses of alternating polarity and increasing amplitude, applied along the [100] arm of the CuMnAs cross device presented in the main manuscript. The location of 180° antiferromagnetic domain walls and their points of chirality reversal are substantially modified with each current pulse. The thick dark and light stripes running along the $[1\bar{1}0]$ and [110] axes in Figure S4a-d are due to the surface termination of a twin defect, a nanometre-scale slab of the crystal which is rotated with respect to the bulk [5]. As described in Ref. [6], these result in a local alignment of the Néel vector in the direction parallel to the stripes. It can be seen that the magnetic contrast due to the twin defects is not affected by the 21 V pulses ($J = 1.2 \times 10^7\,\mathrm{A\,cm^{-2}}$) in Fig. S4a,b, while they are largely erased by the 25 V pulses ($J = 1.5 \times 10^7\,\mathrm{A\,cm^{-2}}$) in Fig. S4e-h.

XMLD-PEEM images of 180° antiferromagnetic domain walls in a 20 µm wide arm oriented along [010] in a second CuMnAs cross device are shown in Fig. S5. In the initial configuration before applying a current pulse (Fig. S5a), it can be seen that 180° walls run approximately in the $[1\bar{1}0]$ direction on either side of a defect stripe, while a third wall runs approximately in the [100] direction. After application of a 1 ms, 90 mA ($1 \times 10^7\,\mathrm{A\,cm^{-2}}$) pulse in the $[0\bar{1}0]$ direction, merons and antimerons are generated, shown in Fig. S5b, localized on the $[1\bar{1}0]$-oriented 180° domain walls. Subsequent 90 mA pulses with alternating polarity cause movement of the 180° domain walls in addition to displacement of the merons and antimerons (Fig. S5c-f).

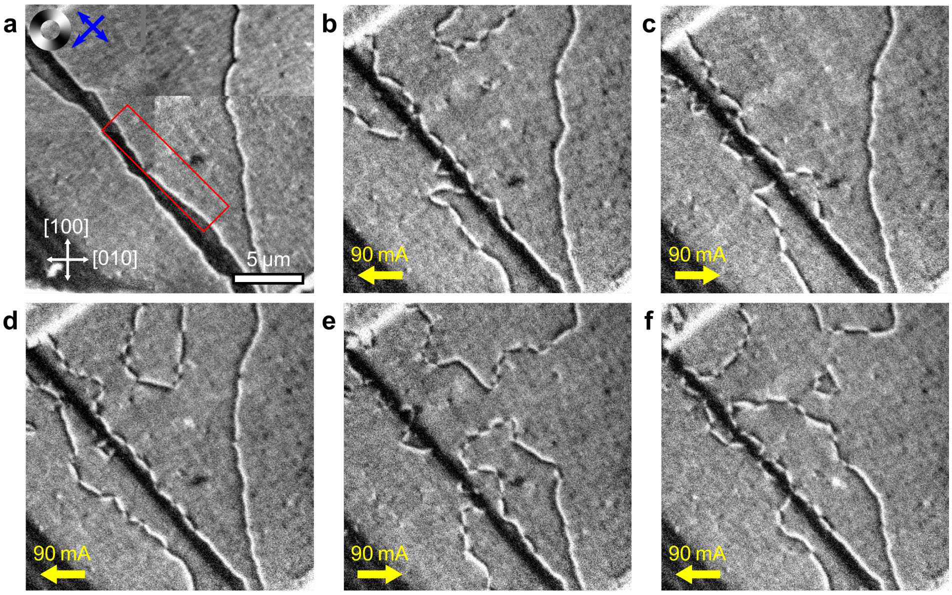

Figure S5: Current-induced generation of merons and antimerons localized on 180° domain walls in a [010] arm of a 20 µm arm width cross device. a, XMLD-PEEM image of the region before pulses were applied. b, After a 1 ms, 90 mA (1×10^7 A cm^{-2}) pulse is applied along the [0$\bar{1}$0] direction, merons and antimerons are created on the domain walls, appearing as points of black/white, white/black chirality reversal. c-f, The same region after applying subsequent 1 ms, 90 mA pulses with alternating polarity.

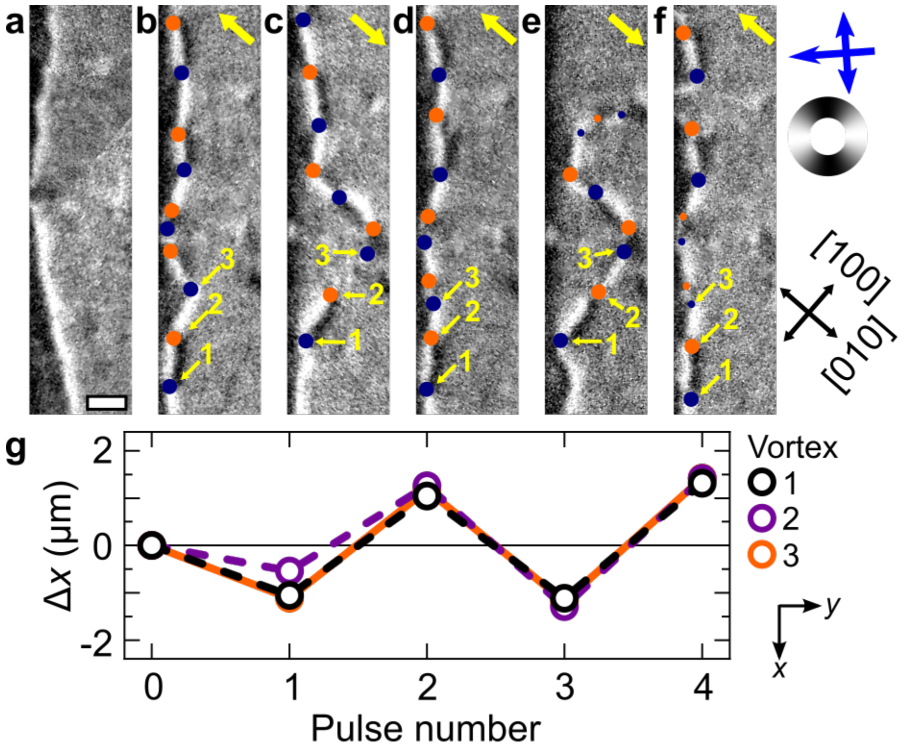

Figure S6: Generation and displacement of merons and antimerons during the sequence of alternating polarity 1 ms, 90 mA pulses. **a**, shows a section of the 180° domain wall before pulsing. The scale bar indicates 1 µm. **b**, shows merons and antimerons (blue and orange circles) generated after the first pulse. **c-f**, a change in their positions during the pulse sequence. **g**, measured vertical displacement, Δx, of merons and antimerons labelled 1-3 during the pulse sequence.

The current-induced displacement of merons and antimerons localised on the domain wall oriented along the $[1\bar{1}0]$ direction is highlighted in Fig. S6. A section of the 180° domain wall, indicated by the red box in Fig. S5a, is shown for the sequence of 1 ms, 90 mA pulses. Fig. S6a shows the domain wall before pulses are applied. After the first pulse, merons and antimerons are formed (Fig. S6b) on the domain wall, with their positions indicated by the blue and orange circles, respectively. The following 90 mA pulses (Fig. S6c-f) cause the merons and antimerons to move in a

reversible way between pinning sites, in a direction determined by the pulse polarity. Figure S6g shows the measured vertical displacement, Δx, of the merons and antimerons labelled 1-3. There is a clear reversible and repeatable behaviour to their displacement, which is attributed to polarity dependent current-driven motion.

Measurements were also performed for the [100] oriented arm of the 20 µm device. Figure S7a shows an XMLD-PEEM image in the initial state, with uniform chirality 180° domain walls running approximately parallel to the twin defects along [110] and $[1\bar{1}0]$. After applying a 1 ms, 90 mA ($1 \times 10^7\,\mathrm{A\,cm^{-2}}$) pulse in the [100] direction, the domain walls have moved, points of chirality reversal on the domain wall have formed (Fig. S7b). Closed domain wall loops have also formed with two points of chirality reversal corresponding to a meron-antimeron pair. The Néel vector variation across the domain wall width for this region of the device, either side of the points of chirality reversal, is presented in section 1.

With the merons and antimerons formed, lower amplitude pulses up to the threshold were applied with alternating polarity (Fig. S7c-g). The displacement of the domain walls is outlined by the red dashed lines in Fig. S7c-g. For current pulses significantly lower than the threshold, merons / antimerons in the domain wall remain pinned while small displacements in the domain wall positions are observed. Closer to the threshold amplitude, at 89 mA, the domain wall is seen to pass through the domain wall loops (Fig. S7f). A new domain wall loop is formed, at a position corresponding to the previous location of a meron / antimeron pair in the domain wall. The opposite polarity 89 mA pulse causes the domain wall to pass back through the group of domain wall loops, and again a new loop is formed (Fig. S7g). Finally, applying a 92 mA pulse along the $[\bar{1}00]$ direction completely changes the position and number of domain walls, as shown in Fig. S7h, and generates a large number of merons and antimerons.

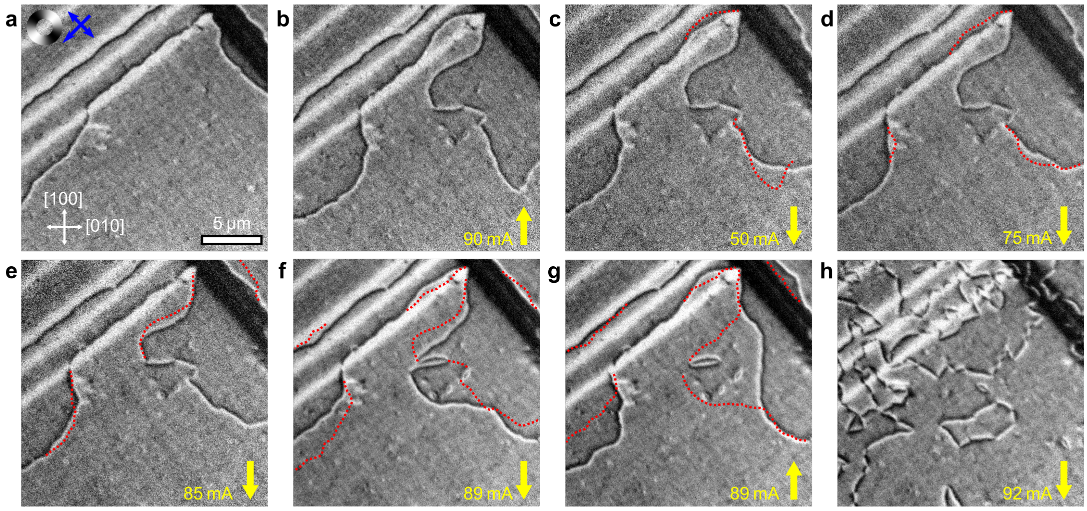

Figure S7: Current-induced generation of merons and antimerons localized on 180° domain walls in a [100] arm of a 20 µm arm width cross device. **a**, Image of the initial domain wall state. **b**, After applying a 1 ms, 90 mA ($1 \times 10^7\,\mathrm{A\,cm^{-2}}$) pulse along the [100] direction. **c-g**, Subsequent pulses of alternating polarity and reduced amplitude cause sections of the domain walls to move and the formation of two new closed loop domain wall structures (f,g). **h**, Applying a 1 ms, 92 mA current pulse along the [$\bar{1}$00] direction completely alters the domain wall state and leads to the formation of a significant number of merons and antimerons.

References

[1] P Wadley, KW Edmonds, MR Shahedkhah, RP Campion, BL Gallagher, J Železný, J Kuneš, V Novák, T Jungwirth, V Saidl, et al. Control of antiferromagnetic spin axis orientation in bilayer Fe/CuMnAs films. *Sci. Rep.*, 7(1):1–6, 2017.

[2] Hariom Jani, Jheng-Cyuan Lin, Jiahao Chen, Jack Harrison, Francesco Maccherozzi, Jonathon Schad, Saurav Prakash, Chang-Beom Eom, Ariando Ariando, Thirumalai Venkatesan, et al. Antiferromagnetic half-skyrmions and bimerons at room temperature. *Nature*, 590(7844):74–79, 2021.

[3] P Wadley, V Hills, MR Shahedkhah, KW Edmonds, RP Campion, V Novák, B Ouladdiaf, D Khalyavin, S Langridge, V Saidl, et al. Antiferromagnetic structure in tetragonal CuMnAs thin films. *Sci. Rep.*, 5(1):1–6, 2015.

[4] M Wang, C Andrews, S Reimers, OJ Amin, P Wadley, RP Campion, SF Poole, J Felton, KW Edmonds, BL Gallagher, et al. Spin flop and crystalline anisotropic magnetoresistance in CuMnAs. *Phys. Rev. B*, 101(9):094429, 2020.

[5] Filip Krizek, Zdeněk Kašpar, Aliaksei Vetushka, Dominik Kriegner, Elisabetta M Fiordaliso, Jan Michalicka, Ondřej Man, Jan Zubáč, Martin Brajer, Victoria A Hills, et al. Molecular beam epitaxy of CuMnAs. *Phys. Rev. Mater.*, 4(1):014409, 2020.

[6] Sonka Reimers, Dominik Kriegner, Olena Gomonay, Dina Carbone, Filip Krizek, Vit Novák, Richard P Campion, Francesco Maccherozzi, Alexander Björling, Oliver J Amin, et al. Defect-driven antiferromagnetic domain walls in CuMnAs films. *Nat. Commun.*, 13(1):1–7, 2022.
