# Supplementary figures and images for: Antiferromagnetic half-skyrmions electrically generated and controlled at room temperature
Source: Nat Nanotechnol. 2023 May 8;18(8):849–53. doi: 10.1038/s41565-023-01386-3 (PMC10427425; doi:10.1038/s41565-023-01386-3)

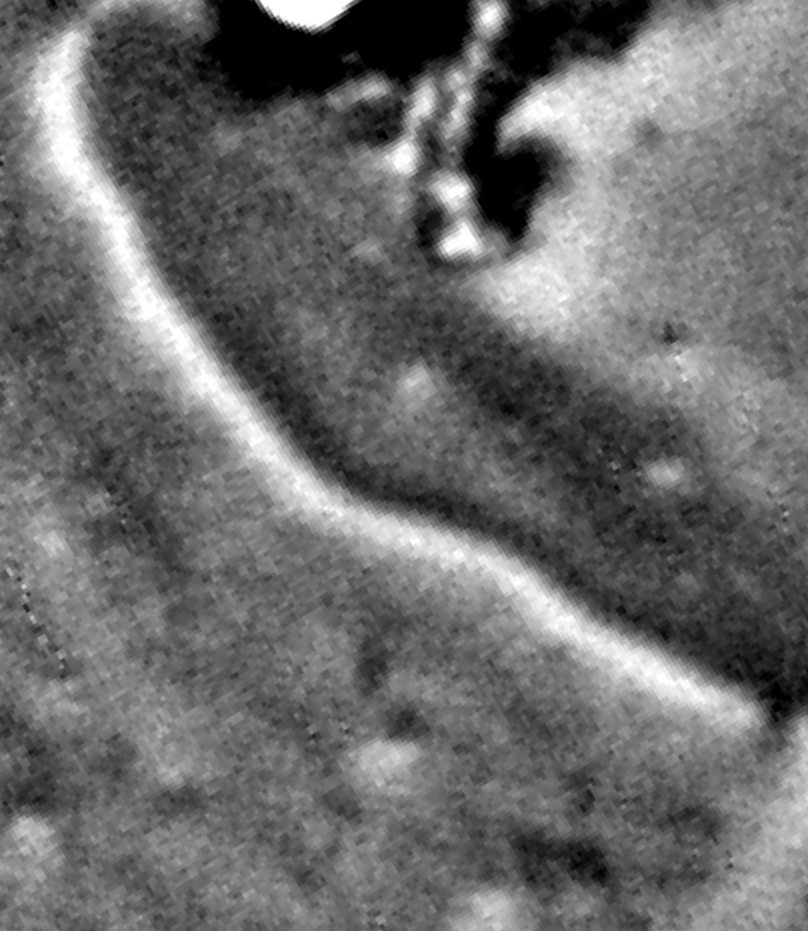

Supplement: Source Data Fig. 2 — Unprocessed PEEM images of AF meron–antimeron pair generation. [file 41565_2023_1386_MOESM2_ESM.zip › fig2/a.tiff]

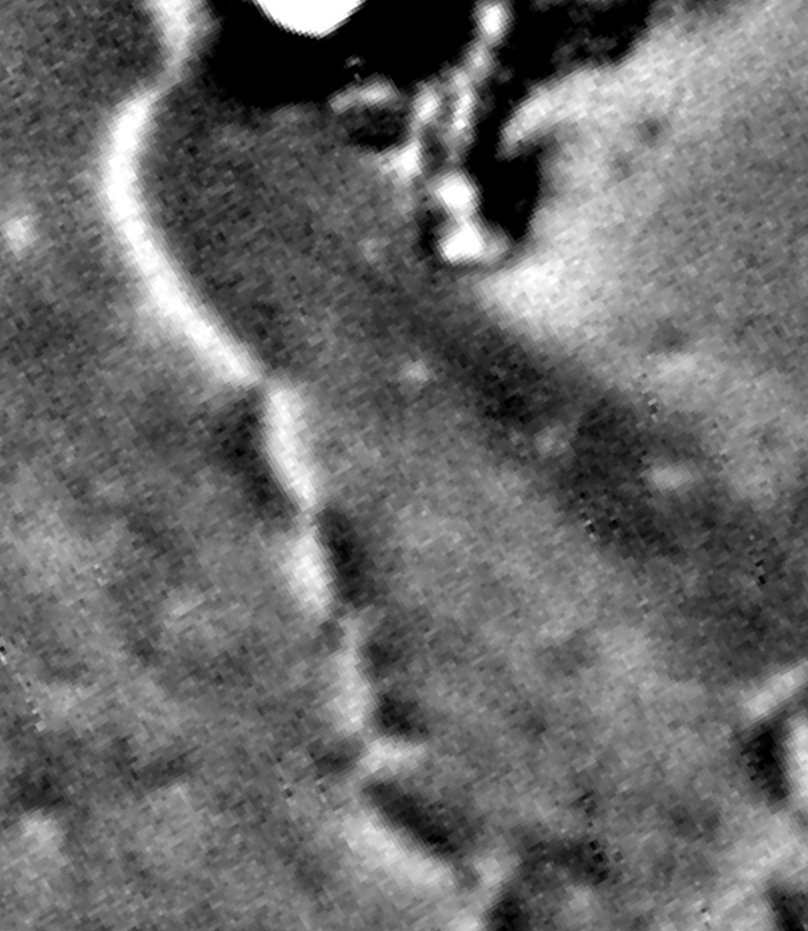

Supplement: Source Data Fig. 2 — Unprocessed PEEM images of AF meron–antimeron pair generation. [file 41565_2023_1386_MOESM2_ESM.zip › fig2/b.tiff]

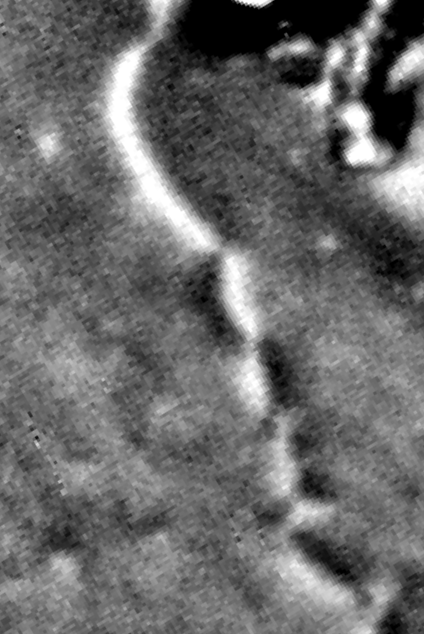

Supplement: Source Data Fig. 3 — Unprocessed PEEM images of current-driven meron–antimeron displacement, and corresponding statistical source data. [file 41565_2023_1386_MOESM3_ESM.zip › fig3/a.tiff]

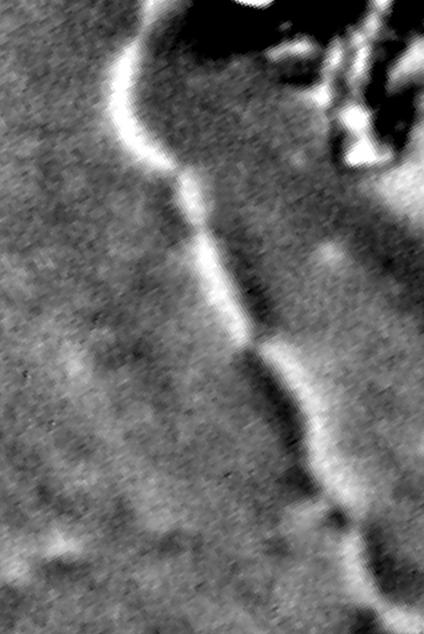

Supplement: Source Data Fig. 3 — Unprocessed PEEM images of current-driven meron–antimeron displacement, and corresponding statistical source data. [file 41565_2023_1386_MOESM3_ESM.zip › fig3/b.tiff]

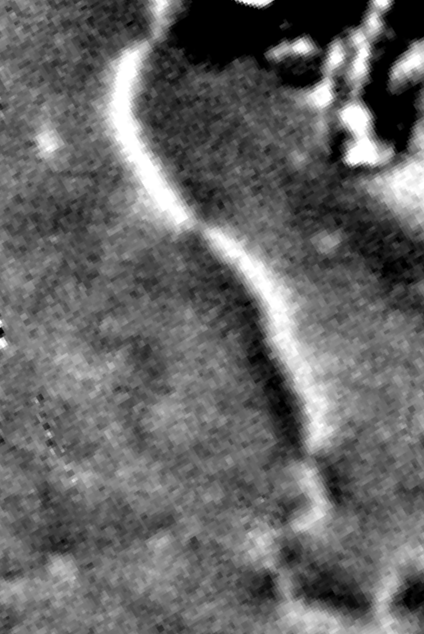

Supplement: Source Data Fig. 3 — Unprocessed PEEM images of current-driven meron–antimeron displacement, and corresponding statistical source data. [file 41565_2023_1386_MOESM3_ESM.zip › fig3/c.tiff]

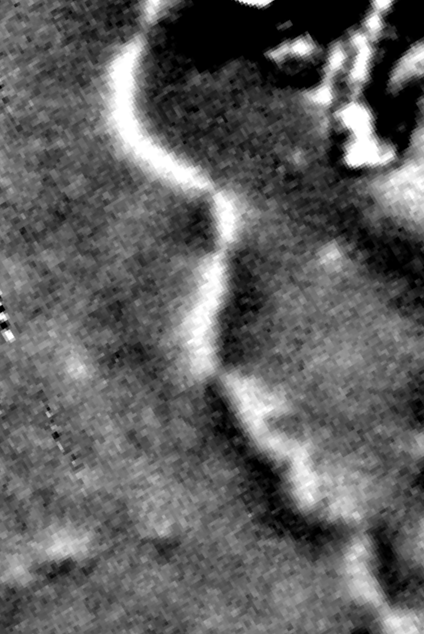

Supplement: Source Data Fig. 3 — Unprocessed PEEM images of current-driven meron–antimeron displacement, and corresponding statistical source data. [file 41565_2023_1386_MOESM3_ESM.zip › fig3/d.tiff]

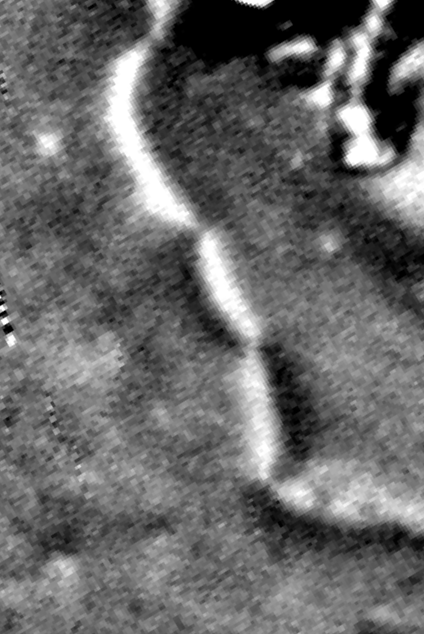

Supplement: Source Data Fig. 3 — Unprocessed PEEM images of current-driven meron–antimeron displacement, and corresponding statistical source data. [file 41565_2023_1386_MOESM3_ESM.zip › fig3/e.tiff]

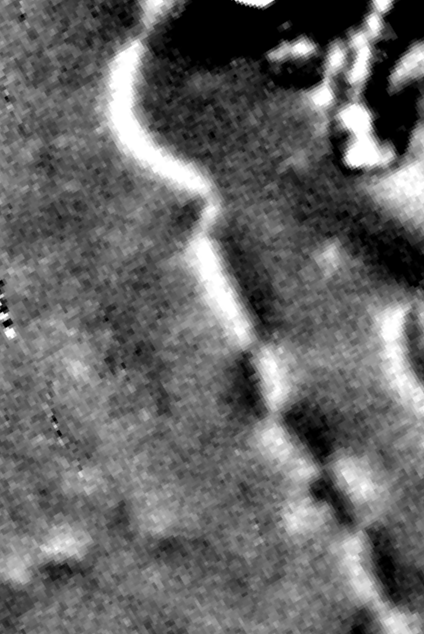

Supplement: Source Data Fig. 3 — Unprocessed PEEM images of current-driven meron–antimeron displacement, and corresponding statistical source data. [file 41565_2023_1386_MOESM3_ESM.zip › fig3/f.tiff]

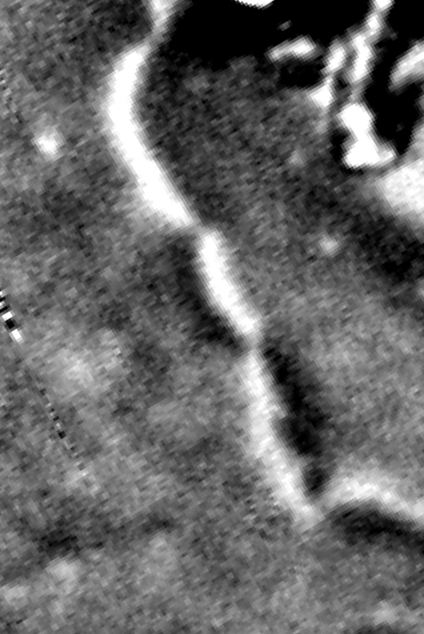

Supplement: Source Data Fig. 3 — Unprocessed PEEM images of current-driven meron–antimeron displacement, and corresponding statistical source data. [file 41565_2023_1386_MOESM3_ESM.zip › fig3/g.tiff]

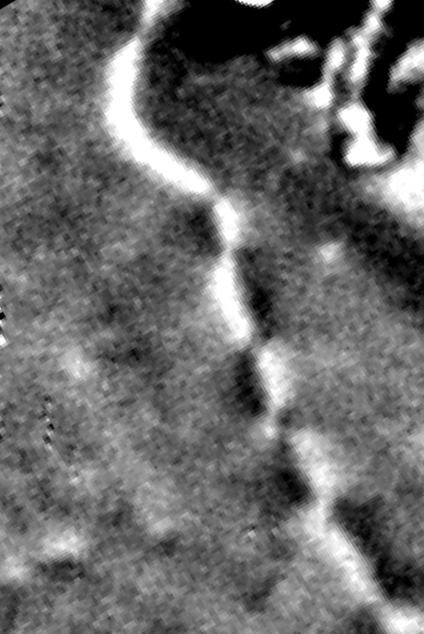

Supplement: Source Data Fig. 3 — Unprocessed PEEM images of current-driven meron–antimeron displacement, and corresponding statistical source data. [file 41565_2023_1386_MOESM3_ESM.zip › fig3/h.tiff]
